# Comparative phylogenetic, antimicrobial resistance, and clinical characterization of human spondylodiscitis-associated *Staphylococcus pseudintermedius*

**DOI:** 10.3389/fmicb.2026.1735075

**Published:** 2026-02-17

**Authors:** Jakob Douan, Christian Kohler, Lyubomir Haralambiev, Evgeny A. Idelevich, Karsten Becker

**Affiliations:** 1 Friedrich Loeffler-Institute of Medical Microbiology, University Medicine Greifswald, Greifswald, Germany; 2 Center for Orthopaedics, Trauma Surgery and Rehabilitation Medicine, University Medicine Greifswald, Greifswald, Germany; 3 Institute of Medical Microbiology, University Hospital Münster, Münster, Germany

**Keywords:** cgMLST, One Health, SNP, spondylodiscitis, *Staphylococcus pseudintermedius*, WGS

## Abstract

We report a case of spondylodiscitis caused by methicillin-susceptible *Staphylococcus pseudintermedius* (MSSP) in a 23-year-old male following lumbar spine stabilization. Despite initial recovery, the patient developed postoperative infection with elevated inflammatory markers and radiological signs of spondylodiscitis. Revision surgery revealed pus extending to the osteosynthesis device. *S. pseudintermedius* was identified from tissue and blood cultures by MALDI-TOF MS and molecular methods. Whole-genome sequencing (WGS) of three isolates collected at different time points revealed a single clonal strain carrying multiple chromosomal resistance genes [*blaZ*, *cat*, *ermB*, *aph*-Stph, *ant6*, *aph*(3″)-III, *sat4A*] and a 3.1 kb plasmid of unknown function, but no *mecA*. Phenotypically, the isolate was susceptible to all tested antibiotics except erythromycin and exhibited inducible clindamycin resistance. Therapy began with clindamycin, later switched to daptomycin, followed by oral levofloxacin and rifampicin, achieving clinical resolution. To contextualize the isolate within the species’ antimicrobial resistance (AMR) landscape, we compared its AMR gene profile with 5,500 publicly available *S. pseudintermedius* genomes. Thirty-four AMR genes were detected, most frequently *aac6-aph2*, *ant6*, *aph2*, *sat*, *aph*-Stph, *blaZ*, *mecA*, *erm*, *tetM*/*tetO*, *cat*, and *dfr*. Cluster analysis revealed three AMR groups: highly multidrug-resistant (clusters 1–2), intermediate (clusters 3–7), and low-AMR (clusters 8–10). Our isolate fell into cluster 7, enriched for aminoglycoside, *β*-lactam, macrolide, tetracycline, and phenicol resistance genes. Overall, 42.5% of genomes carried multidrug-resistant gene constellations, whereas 57.5% harbored few AMR genes, with *mecA* rare in low-AMR clusters. Virulence profiling of our isolate indicated diverse toxins, adhesion factors, biofilm-related autolysins, and immune evasion proteins, supporting pathogenic potential. Phylogenetic analysis using MLST and core-SNPs demonstrated high genomic diversity among *S. pseudintermedius* worldwide. HGW2412 belonged to the rare sequence type ST2051, previously reported only in Poland. Despite clustering with isolates from multiple continents, precise geographic inference was limited. This case highlights the value of WGS and advanced molecular diagnostics for managing *S. pseudintermedius* infections and underscores the need for standardized surveillance within a One Health framework.

## Introduction

Spondylodiscitis encompasses vertebral osteomyelitis, spondylitis, and discitis as variations of a shared pathological process (Gouliouris et al., 2010). Among pyogenic causes, *Staphylococcus aureus* is most frequently isolated in humans (Mylona et al., 2009), whereas *Staphylococcus pseudintermedius* is the predominant agent in dogs (Barash et al., 2018; Pilkington et al., 2023). Staphylococci are known for antibiotic resistance and diverse virulence factors and are roughly classified for clinical purposes by coagulase production (Kloos and Bannerman, 1994). Importantly, *S. pseudintermedius*, a coagulase-positive species, is primarily part of the canine flora (Bannoehr and Guardabassi, 2012), yet it causes infections in companion but also wild animals (Smith et al., 2020; Mama et al., 2019). Though mainly an animal pathogen, it can act as a zoonotic agent with rare human cases mostly among those in close contact with animals (Darlow et al., 2017; Morris et al., 2010; Schwartz et al., 2021; Tugasworo et al., 2021). The first human case published with this species name was reported in 2005 involving an infected defibrillator implant (Van Hoovels et al., 2006), but, it can be assumed that earlier cases occurred under the designation *S. intermedius*.

Formally delimited from *S. intermedius* in 2005 (Devriese et al., 2005), *S. pseudintermedius* is phylogenetically part of the *S. intermedius* group (SIG) and was in the pre-MALDI-TOF mass spectrometry era frequently confused with other SIG species or misidentified as *S. aureus* due to coagulase activity (Becker et al., 2005; Fitzgerald, 2009; Sasaki et al., 2007). Misidentification may affect the interpretation of susceptibility testing and treatment decisions (Borjesson et al., 2015). *S. pseudintermedius* produces various virulence factors and, like other staphylococci, presents challenges through methicillin resistance (Bannoehr et al., 2011; Piriz et al., 1996; Pitchenin et al., 2018; Roberts et al., 2024; Teixeira et al., 2024). Further, methicillin-resistant *S. pseudintermedius* (MRSP) isolates are often multidrug-resistant and pose significant zoonotic risks (Kadlec and Schwarz, 2012; Paul et al., 2011).

Infections caused by *S. pseudintermedius* are increasingly recognized as clinically similar to those caused by *S. aureus*, particularly in vulnerable populations such as the elderly and immunocompromised (Somayaji et al., 2016). A 2017 review identified MRSP as one of the most common antimicrobial-resistant (AMR) bacteria transmitted from companion animals to humans (Pomba et al., 2017). In Germany, individuals aged 60 and older represent 25% of pet owners (Schirmer, 2018), and their close contact with pets may elevate the risk of zoonotic transmission of pathogens like *S. pseudintermedius*. This underscores the necessity for targeted surveillance and preventive measures within this demographic and beyond. Further emphasizing its public health relevance, the European Food Safety Authority’s Panel on Animal Health and Welfare named *S. pseudintermedius* among significant AMR pathogens isolated from dogs in the EU, alongside *Escherichia coli* and *Pseudomonas aeruginosa* (Nielsen et al., 2021). The misuse of antimicrobials shared between companion animals and humans exacerbates this issue (Chow et al., 2024). These findings align with global health research advocating for integrated surveillance systems to monitor AMR in zoonotic bacteria, including *Staphylococcus* species, under the One Health framework (Dafale et al., 2020).

Here, we report a rare case of spondylodiscitis caused by *S. pseudintermedius* in a young male with no known animal contact following spinal trauma and surgery. The isolates recovered underwent whole-genome sequencing (WGS) and genetic characterization to enable phylogenetic classification and to identify virulence factors and resistance genes. This case highlights the importance of recognizing zoonotic pathogens within a “One Health” framework and emphasizes the need for precise and advanced diagnostic techniques.

## Materials and methods

### Patient history

**Figure 1:**
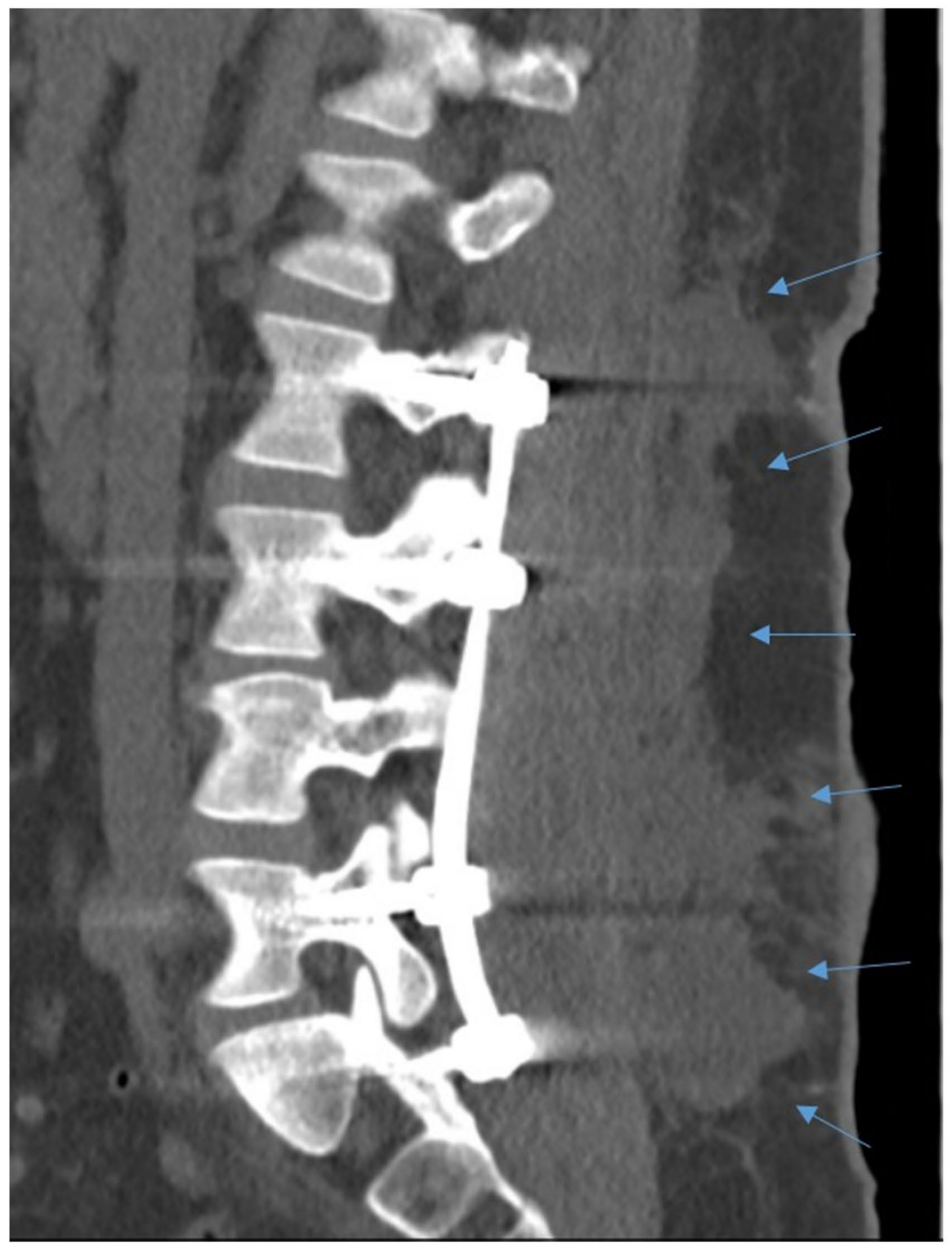
CT scan of spondylodiscitis in a 23-year-old patient following surgical stabilization of L2–L5. The CT image reveals substantial fluid accumulation surrounding the internal transpedicular osteosynthesis of the lumbar spine, signifying early postoperative infection. The arrows mark the soft tissue involvement. The imaging findings are consistent with the patient’s septic clinical presentation, thereby confirming the suspected diagnosis of a peri-implant abscess.

A 23-year-old male patient with a history of Tourette syndrome and a documented penicillin allergy presented to the emergency department after sustaining a traumatic fall from a second-story balcony. Upon admission, the patient reported severe pain in the cervical, thoracic, and lumbar regions, with additional discomfort in the thorax and both ankles. The initial examination revealed an unstable compression fracture of the fourth lumbar vertebra without evidence of an open wound or external communication. Imaging confirmed the lumbar fracture without additional injuries to the head, thorax, or abdomen. The patient was admitted for surgical treatment of the spinal fracture and underwent transpedicular dorsal stabilization of L2 to L5 at the end of September 2024. The postoperative course was initially uneventful, and the patient demonstrated adequate recovery. However, despite a sudden deterioration in the patient’s infection markers, he was discharged against medical advice 11 days after admission, without undergoing any antibiotic treatment. Three days after being discharged, the patient presented at a nearby hospital with worsening general condition and progressive pain. As a result, the patient was readmitted to our clinic for further treatment. Upon readmission, the patient exhibited signs of infection, including fever and elevated inflammatory markers, with a C-reactive protein (CRP) level of 282 mg/L and leukocytosis with a white blood cell count 11.9 × 10^9^/L. A series of blood cultures were collected for microbiological examination. Further, a computed tomography (CT) scan was performed, revealing signs of spondylodiscitis at the L4 vertebral level (Figure 1). Consequently, the patient underwent revision surgery, which revealed pus in the wound extending to the osteosynthesis. Following meticulous surgical debridement and lavage, five tissue samples were obtained from different areas. The transpedicular osteosynthesis was retained as the fracture had not yet consolidated.

### Microbiological analysis

Bacterial growth was observed after overnight cultivation of the tissue samples, with colonies appearing white and haemolytic on Columbia agar plates with 5% sheep blood (BD Diagnostics, Heidelberg, Germany). All tested colonies were positive in the catalase test, while the clumping factor test using the Pastorex Staph-Plus (Bio-Rad, Marnes-la-Coquette, France) was negative. Identification of the bacterial colonies from these samples was performed using MALDI-TOF MS utilizing the MALDI Biotyper® sirius system (Bruker Daltonics, Bremen, Germany) with MBT Biotargets 96 (Bruker Daltonics). Identification was initially performed using two protocols, direct transfer (DT) and extended direct transfer (eDT), following the manufacturer’s instructions and a previous study (Letunic and Bork, 2024). Some spots showed no peaks or identification scores below 2.00 and therefor the median identification score was 1.70 (range = 1.60–2.12). To improve sensitivity and accuracy, a protein extraction (PE) procedure was applied before repeating MALDI-TOF MS measurements (Idelevich et al., 2023). This resulted in a marked increase in the identification scores, with a median value of 2.22 (range = 2.08–2.37), and all isolates were unequivocally identified as *S. pseudintermedius*. For quality control and standardization, a 1 μL aliquot of IVD Bacterial Test Standard (IVD BTS, Bruker) was included on the MBT Biotarget during all three procedures, per manufacturer guidelines.

Of the six-vial blood culture set initially collected, only one aerobic vial (BACTEC™ Plus; BD Diagnostics) tested positive. Gram staining showed gram-positive cocci consistent with staphylococci. This vial was analyzed using the loop-mediated isothermal amplification-based eazyplex® MRSA kit (Amplex Diagnostics), which detects *S. aureus* complex species (*S. aureus, S. argenteus, S. schweitzeri, S. roterodami*), *S. epidermidis*, and *mecA*/*mecC* resistance genes. No target genes were detected in this case. The positive blood culture vial was subcultured on Columbia blood agar (BD Diagnostics, Heidelberg, Germany), yielding white, hemolytic colonies similar to those from tissue samples, both negative for clumping factor (Pastorex Staph-Plus, Bio-Rad, Marnes-la-Coquette, France). After protein extraction procedure (PE), MALDI-TOF MS identified all isolates from tissue and blood cultures as *S. pseudintermedius* with median score of 2.22 (range = 2.11–2.28). To exclude contamination, two additional blood culture sets were collected earlier; one aerobic vial was positive, and its colonies were identified as *S. pseudintermedius*. Except for these two positive aerobic vials, all other blood cultures remained negative after 6 days of incubation. No other bacteria or fungi were detected in clinical or screening samples throughout treatment.

The antimicrobial susceptibility testing (AST) of the isolates was performed using the VITEK 2 system (bioMérieux, Marcy l’Etoile, France) and VITEK software (Version 9.04) following the manufacturer’s instructions with the AST-P-654 test cards. Phenotypic methicillin resistance was further assessed by disk diffusion testing using oxacillin (1 μg), as recommended by the European Committee on Antimicrobial Susceptibility Testing (EUCAST) for screening in *S. pseudintermedius*, *S. intermedius*, *S. schleiferi,* and *S. coagulans*. AST results were interpreted according to the EUCAST Breakpoint Tables for MICs and zone diameters, version 14.0. All isolates were found to be susceptible to all tested antibiotics, except for erythromycin and clindamycin (Table 1).

**Table 1:** Antimicrobial susceptibility characteristics of *Staphylococcus pseudintermedius* strain HGW2412.

Antibiotics	Minimum inhibitory concentrations (MICs), mg/L	Interpretation^a^
Oxacillin	<=0.25	Susceptible (S)
Levofloxacin	<=0.12	No interpretation
Gentamicin	<=0.5	No interpretation
Teicoplanin	1	No interpretation
Vancomycin	<=0.5	No interpretation
Erythromycin	> = 8	Resistance (R)
Clindamycin	0.25	Inducible clindamycin resistance (R)
Tetracycline	<=1	Susceptible (S)
Tigecycline	<=0.12	Susceptible (S)
Linezolid	1	Susceptible (S)
Daptomycin	0.25	Susceptible (S)
Fosfomycin	<=8	Susceptible (S)
Fusidic acid	<=0.5	Susceptible (S)
Trimethoprim sulfamethoxazole	<=10	Susceptible (S)

^a^European Committee on Antimicrobial Susceptibility Testing (EUCAST, 2019) definition: S = Susceptible (standard dosing regimen), I = Susceptible (increased exposure), R = Resistant.

### DNA isolation, library preparation and whole genome sequencing

Bacteria were cultured on blood agar plates (Becton, Dickinson and Company [BD], USA) at 37 °C for 16–24 h. Cells were harvested using a 1 μL inoculation loop, and genomic DNA was extracted with the NucleoSpin® Microbial DNA Kit (Macherey-Nagel, Germany) according to the manufacturer’s instructions. DNA purity and quality were assessed using the NanoDrop™ One/OneC Spectrophotometer (Thermo Fisher Scientific, USA), while DNA concentrations were determined using the Qubit™ 1X dsDNA Assay Kit and Qubit™ 4 Fluorometer (Thermo Fisher Scientific, USA). DNA was either used immediately for library preparation or stored at −80 °C until further processing.

Due to the use of two different sequencing platforms, separate library preparation protocols were followed. For Illumina short-read sequencing, libraries were prepared using the Illumina DNA Prep Kit following the manufacturer’s instructions, and sequencing was performed on a MiSeqDx system (Illumina, USA) using a 2 × 300 bp paired-end configuration.

For Oxford Nanopore (ONT) long-read sequencing, libraries were prepared using the Rapid Barcoding Kit SQK-RBK114.24 (Oxford Nanopore Technologies, UK) as per the manufacturer’s protocol. Sequencing was conducted using a MinION device equipped with an R10.4.1 flow cell (FLO-MIN114).

### Assembly and polishing of consensus sequences

Raw Illumina reads were processed using the platform’s default basecalling settings, and the resulting FASTQ files were subsequently used for hybrid assemblies. For Oxford Nanopore Technologies (ONT) sequencing, raw signal data (POD5 files) were basecalled using the super-accurate (SUP) model dna_r10.4.1_e8.2_400bps_sup@v5.0.0 with the Dorado v0.9.1 basecaller^1^. The resulting ONT FASTQ reads were first used for genome size estimation with Long Read-based Genome size Estimation (LRGE) (Hall et al., 2024), followed by downsampling to 100 × coverage using Rasusa (Randomly Subsample Sequencing Reads or Alignments) (Hall, 2022). Downsampled FASTQ files were then assembled using the Autocycler pipeline v0.2.1 in fully automated mode, utilizing the following long-read assemblers: Flye, Miniasm, NextDenovo, and Raven. Resulting consensus sequences were polished with Medaka v2.0.1, applying the bacterial methylation model r1041_e82_400bps_bacterial_methylation^2^. Polished assemblies were reoriented using Dnaapler (Bouras et al., 2024), aligning sequences to start at the *dnaN* gene for chromosomes or the *rep* gene for plasmids, thereby standardizing orientation across all sequenced isolates. Final assemblies were hybrid-polished using high-quality Illumina short reads. These reads were quality-trimmed with Trimmomatic v0.39, and final polishing was performed using Polypolish v0.6.0 (Bouras et al., 2024; Wick and Holt, 2022; Bolger et al., 2014). The resulting high-quality consensus sequences were used in all downstream analyses.

### Genome annotation, comparative genomics and phylogenetic analyses

Initially, the consensus sequences were analyzed using the TORMES pipeline v1.3.0^3^ (Quijada et al., 2019) for genome annotation via Prokka (Seemann, 2014) and identification of antimicrobial resistance genes using the CARD, ResFinder, and ARG-ANNOT databases (McArthur et al., 2013; Zankari et al., 2012; Gupta et al., 2014) (Supplementary Table S1). In addition, the sequences of *S. pseudintermedius* HGW2412 and pHGW2412 were annotated using the Bakta pipeline^4^ (Schwengers et al., 2021) (Supplementary Table S1).

Subsequently, Prokka-annotated sequences were employed for virulence gene identification. To this end, the complete protein dataset of the Virulence Factor Database (VFDB^5^) was downloaded, and *Staphylococcus*-specific virulence factors were extracted. These were used in a BLAST+ (v2.16.0+) search^6^ against the HGW2412 proteome. Hits were filtered using the following thresholds (Supplementary Table S1): alignment coverage (= align_l/s_end) ≥ 50%, sequence identity ≥ 30%, and bit score ≥ 100.

To assess the phylogenetic context of HGW2412, 5,500 *S. pseudintermedius* genomes were retrieved from the NCBI Reference Sequence Database (RefSeq)^7^. Pairwise genomic similarity was computed using the Fast Average Nucleotide Identity algorithm (FastANI)^8^ (Jain et al., 2018), and the 100 most closely related genomes were selected for downstream analysis.

These 100 genomes, along with HGW2412, were first subjected to MLST profiling in SeqSphere+ v10.5.0 using the Solyman et al. (2013) scheme, and a minimum spanning tree was constructed.

In parallel, core SNP-based phylogenetic analysis was conducted. An optimal k-mer size of 17 was determined using Kchooser4, and SNP calling was performed with kSNP4.1 (Hall and Nisbet, 2023). A maximum likelihood (ML) phylogenetic tree was then inferred from the core SNP matrix using RAxML v8.2.12 (Stamatakis, 2014), applying the GTRGAMMA substitution model and 1,000 bootstrap replicates. The annotated ML tree, including information on isolation source, country, and bootstrap values, was visualized using Interactive Tree of Life (iTOL v7.1.1) (Letunic and Bork, 2024).

### AMR gene profiling

**Figure 2:**
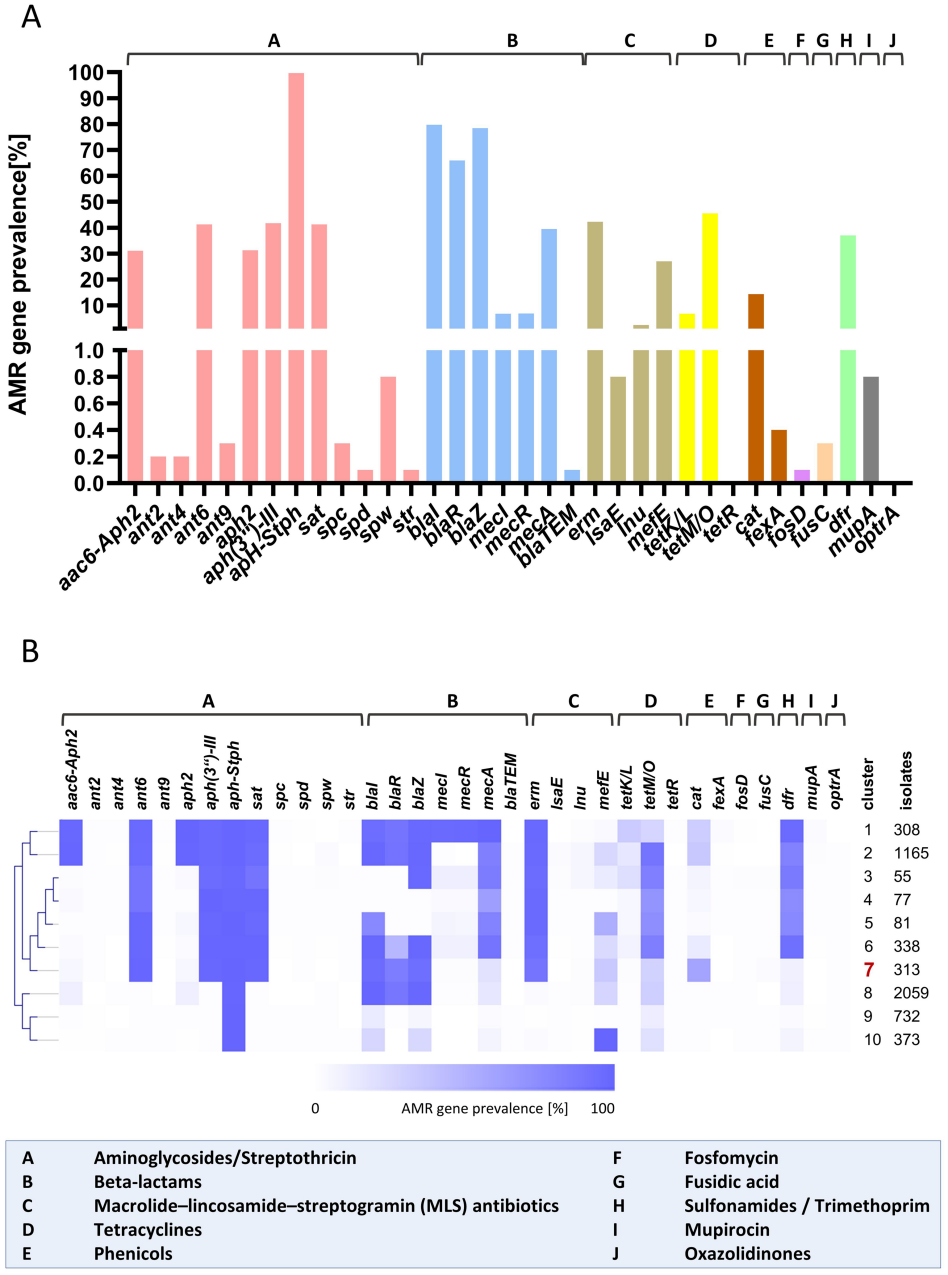
AMR gene prevalence and cluster analysis of *S. pseudintermedius* genomes. **(A)** A total of 5,500 *S. pseudintermedius* genomes from the NCBI RefSeq database, together with the HGW2412 genome, were screened for AMR determinants using ABRicate v1.0.1 (https://github.com/tseemann/abricate). AMR gene prevalences were calculated across all genomes. Colors and letter codes denote antibiotic classes to facilitate interpretation. **(B)** Presence–absence profiles of all detected AMR genes were used as input for clustering with the Self-Organizing Tree Algorithm (SOTA). The resulting hierarchical structure is shown alongside AMR gene prevalence patterns for each cluster, enabling comparison of resistance gene compositions across clusters. Cluster identifiers and the number of genomes per cluster are indicated. The HGW2412 isolate is assigned to Cluster 7 and highlighted accordingly. Figures were generated using GraphPad Prism v9.5.1 (GraphPad Software, USA) and MeV v4.9.0 (TM4 Software Suite, USA). *aac6-aph2*, Aminoglycoside N-acetyltransferase; *ant2*, Aminoglycosid 2′-phosphotransferase; *ant4*, Aminoglycosid 4′-phosphotransferase; *ant6*, Aminoglycosid 6′-phosphotransferase; *ant9*, Aminoglycosid 9′-phosphotransferase; *aph2*, Aminoglycoside 2″-phosphotransferase; *aph(3″)-III*, Aminoglycoside 3″-phosphotransferase III; *aph-Stph*, Aminoglycoside phosphotransferase from *Staphylococcus* species; *sat*, Spectinomycin acetyltransferase; *spc*, Spectinomycin resistance gene; *spd*, Spectinomycin dehydratase; *spw*, Spectinomycin resistance gene; *str*, Streptomycin resistance gene; *blaI*, Beta-lactamase repressor; *blaR*, Beta-lactamase regulatory protein; *blaZ*, Beta-lactamase; *mecI*, Methicillin resistance repressor; *mecR*, Methicillin resistance regulatory protein; *mecA*, Methicillin resistance protein; *blaTEM*, Beta-lactamase TEM; *erm*, Erythromycin methyltransferase; *lsaE*, lincosamide and streptogramin A resistance protein E; *lnu*, Lincosamide nucleotidyltransferase; *mefE*, Macrolide efflux pump; *tetK/L*, Tetracycline efflux pump; *tetM/O*, Tetracycline resistance ribosomal protection protein; *tetR*, Tetracycline resistance repressor; *cat*, Chloramphenicol acetyltransferase; *fexA*, phenicol efflux pump; *fosD*, Fosfomycin resistance gene; *fusC*, Fusidic acid resistance gene; *dfr*, Dihydrofolate reductase; *mupA*, Mupirocin resistance protein; *optrA*, oxazolidinone resistance protein A.

To investigate AMR determinants, a total of 5.500 *S. pseudintermedius* genomes and their associated metadata were downloaded from the RefSeq database (NCBI^9^) as described above. AMR gene detection was performed using ABRicate^10^ with four reference databases: NCBI AMRFinderPlus, CARD, ResFinder, and ARG-ANNOT (Zankari et al., 2012; Gupta et al., 2014; Feldgarden et al., 2019; Jia et al., 2017). Because the different databases can report partially distinct AMR genes, multiple hits corresponding to the same gene within a genome were collapsed into a single entry, ensuring that each AMR gene was counted only once per isolate. The results from all databases were then merged into a unified presence/absence matrix reflecting the similarity of each detected gene to its respective reference sequence (Supplementary Table S2). A minimum sequence identity threshold of 80% was applied, and gene names were standardized by replacing outdated nomenclature with the currently accepted designations. To calculate AMR gene prevalence across the isolates, all hits were binarized (presence = 1 or 100%). This matrix was subsequently used to identify AMR gene clusters employing the Self-Organizing Tree Algorithm (SOTA) implemented in MeV v4.9.0 (TM4 Software Suite, USA) using default settings exceptional the number of cycles which was adjusted to in total 10 cycles. Fewer cycles resulted in overly coarse clustering with high within-cluster variability, whereas more cycles produced overly fine clusters, some of which contained only very few isolates. The SOTA dendrogram depicts the hierarchical relationships among clusters; however, the accompanying heatmap represents relative internal node values rather than true gene prevalence. Therefore, AMR gene prevalence was recalculated for each cluster and visualized as a heatmap adjacent to the dendrogram in Figure 2. The whole ABRicate results table and all matrices (complete heatmap table of 5,501 sequences and individual SOTA clusters 1–10) are provided in Supplementary Table S2.

## Results

### Patient history and microbiological analyses

A 23-year-old man presented with a closed, unstable compression fracture of the fourth lumbar vertebra following a fall from a second-story balcony. He initially underwent transpedicular stabilization from L2 to L5, and his early postoperative recovery was uneventful. Despite elevated infection markers, he left the hospital against medical advice without receiving antibiotics. Three days later, he returned with worsening back pain, fever, and markedly increased inflammatory markers; imaging revealed spondylodiscitis at L4. During revision surgery, purulent material was observed around the osteosynthesis, and five tissue samples were collected for microbiological analysis. Culture of these samples produced white, hemolytic colonies that were catalase-positive but negative for clumping factor. After protein extraction, MALDI-TOF MS confirmed all isolates as *S. pseudintermedius*. Blood cultures obtained during readmission also grew *S. pseudintermedius* in two aerobic vials, while other cultures remained negative. Notably, the patient had no contact with pets either prior to the fall or after discharge, a detail specifically inquired about by the treating physicians given that *S. pseudintermedius* is typically associated with animals. No *mecA* or *mecC* resistance genes were detected, and antimicrobial susceptibility testing indicated that the isolates were susceptible to all tested agents except erythromycin and clindamycin. Collectively, these findings identify *S. pseudintermedius* as the pathogen responsible for the patient’s postoperative spondylodiscitis. Detailed patient history and microbiological analyses information’s are provided in the Materials and Methods section.

### Treatment and outcomes

The *S. pseudintermedius* infection posed a therapeutic challenge due to the patient’s documented penicillin allergy. Although the strain was methicillin-susceptible, beta-lactam antibiotics were contraindicated, necessitating alternative treatment. Empirical therapy with intravenous clindamycin (600 mg TID) was started for 3 days. Following species identification and antimicrobial susceptibility testing, therapy was changed to intravenous daptomycin (8 mg/kg body weight, corresponding to 1,250 mg once daily) for 3 weeks, in accordance with the AST results and the patient’s clinical condition. Five days after the first revision surgery, a second extensive debridement and lavage were performed. The patient responded well, with significant decreases in CRP and leukocyte counts within 2 weeks, no new neurological deficits, and stable follow-up imaging showing no progression of spondylodiscitis. Upon completion of intravenous therapy, the patient was discharged on oral levofloxacin and rifampicin to ensure eradication of residual bacteria, considering their pharmacokinetics and broad-spectrum activity.

At six-week follow-up, the patient was symptom-free with no signs of infection. The transpedicular dorsal spondylodesis was safely removed 6 months later after CT scans confirmed fracture healing.

### Genetic characterization of *S. pseudintermedius* isolates

**Figure 3:**
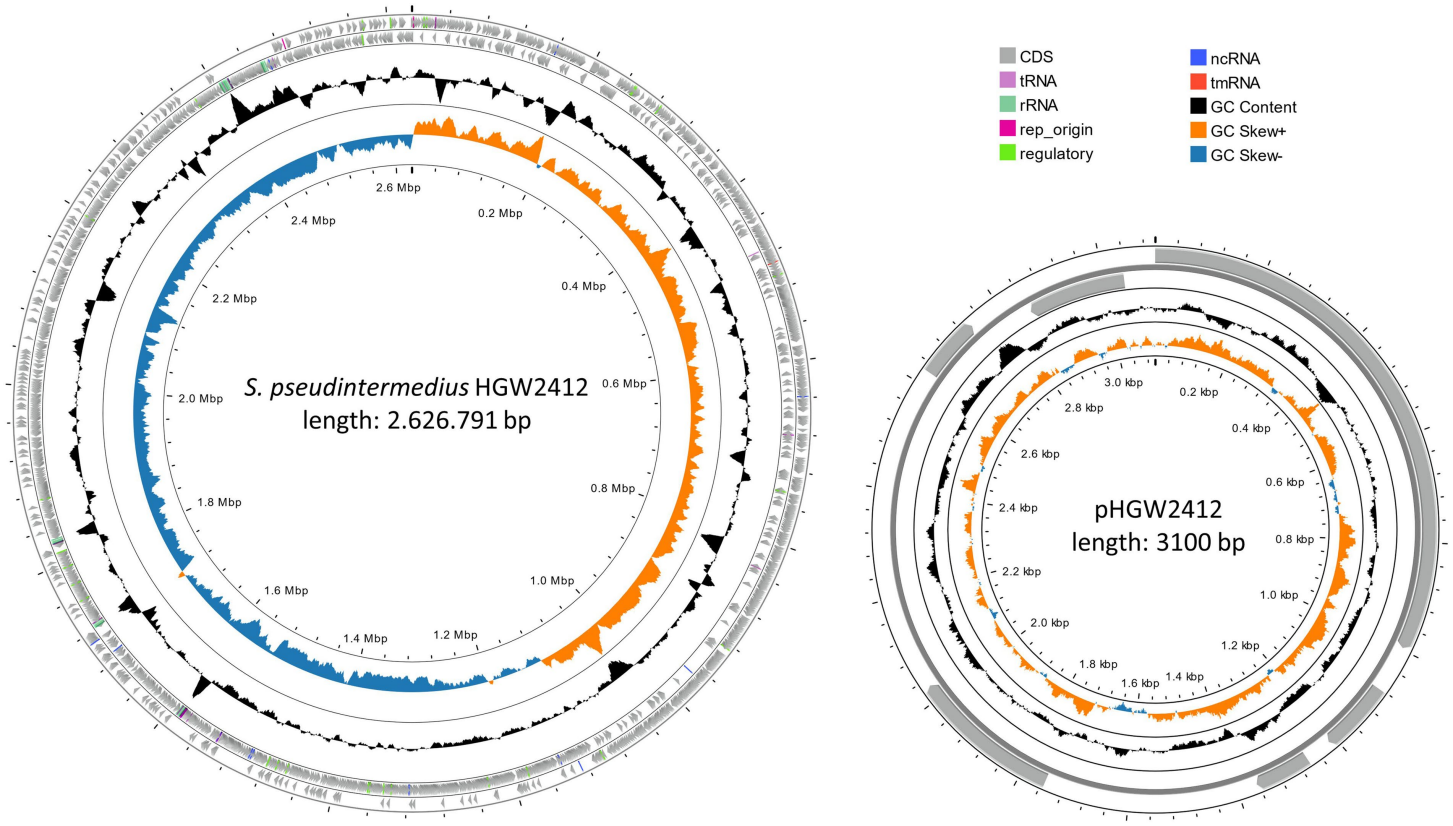
Circular map of the *S. pseudintermedius* HGW2412 chromosome and its plasmid pHGW2412. The innermost circle displays the GC skew (blue and orange), followed by the GC content (black). The next two rings represent the reverse and forward strands, showing the coding DNA sequences (CDS, gray). Also annotated are tRNAs, rRNAs, ncRNAs, tmRNAs, replication origins (rep_origins), and other regulatory sequences. The figure was generated using Proksee (https://proksee.ca/).

To complement the microbiological diagnostics, comprehensive WGS was performed on three *S. pseudintermedius* isolates of the patient. Two isolates were obtained from infected tissue and blood during the initial surgery, and a third isolate was collected 5 days later during wound revision. Hybrid assemblies were generated using both short-read (Illumina) and long-read (Oxford Nanopore Technologies) platforms. All three isolates contained an identical chromosome of 2,626,791 bp and a 3,100 bp plasmid (pHGW2412) (Figure 3). No SNPs or indels were detected between the isolates (data not shown), indicating infection by a single clonal strain. Therefore, all subsequent analyses were conducted using the genome of the initial isolate recovered from tissue.

Initial sequence analysis was performed using the TORMES pipeline (Quijada et al., 2019), including gene annotation with Prokka (Supplementary Table S1 – annotations tabs) and resistance gene screening via ABRicate (Zankari et al., 2012; Gupta et al., 2014; McArthur and Tsang, 2017) (Supplementary Table S1 – antibiotic resistance). The isolate harbored chromosomal genes encoding proteins responsible for resistance to various antibiotics, including *β*-lactams (*blaZ*), chloramphenicol (*cat*), macrolides (*ermB*), and aminoglycosides [*aadE = ant*(*6*)], *aph*-Stph, *aph*(3″)-III, and *sat4A*. Virulence factor screening with the Virulence Factor Database VFDB, see footnote^5^ revealed high-identity protein homologs of *S. aureus* toxins including beta-hemolysin (Hlb, 74.09%), staphylococcal enterotoxin C (Sec, 57.58%), superantigen-like protein SSL11 (Set26, 44.68%), and leukocidins LukS and LukF from *S. intermedius* (98.71 and 99.39% respectively) (Supplementary Table S1). Additional gene homologs were detected for iron acquisition systems (*sfaABCD*, *sbnABCDEFGHI*, and *sirABC*), capsule biosynthesis (*cap8OP*), adhesion (*icaABC*, *vWb*, and *fnbB*), tissue invasion (*lip*, *geh*, *nuc*, and *aur*), and immune evasion (*sbi*) (Supplementary Table S1). Furthermore, an autolysin gene homolog (*aae*) has been detected. Besides other functions, autolysins play a pivotal role in adherence and biofilm formation and have been described for several species such as *S. aureus* and various coagulase-negative staphylococci (Hussain et al., 2015; Heilmann et al., 1997; Biswas et al., 2006). The mere presence of homologs to these virulence factors suggests a considerable virulence potential of this isolate, which was supported by the course and severity of the infection.

The plasmid pHGW2412 encodes six distinct proteins, none of which resemble known antibiotic resistance determinants. Two proteins were associated with plasmid replication (Rep_1 and a mobilization protein) (Supplementary Table S1 – annotation pHGW2412). BLAST analyses revealed that pHGW2412-like sequences occur in other *S. pseudintermedius* genomes, often fragmented and unannotated, likely due to older, less accurate assembly methods. However, the exact biological function of the plasmid remains unknown.

**Figure 4:**
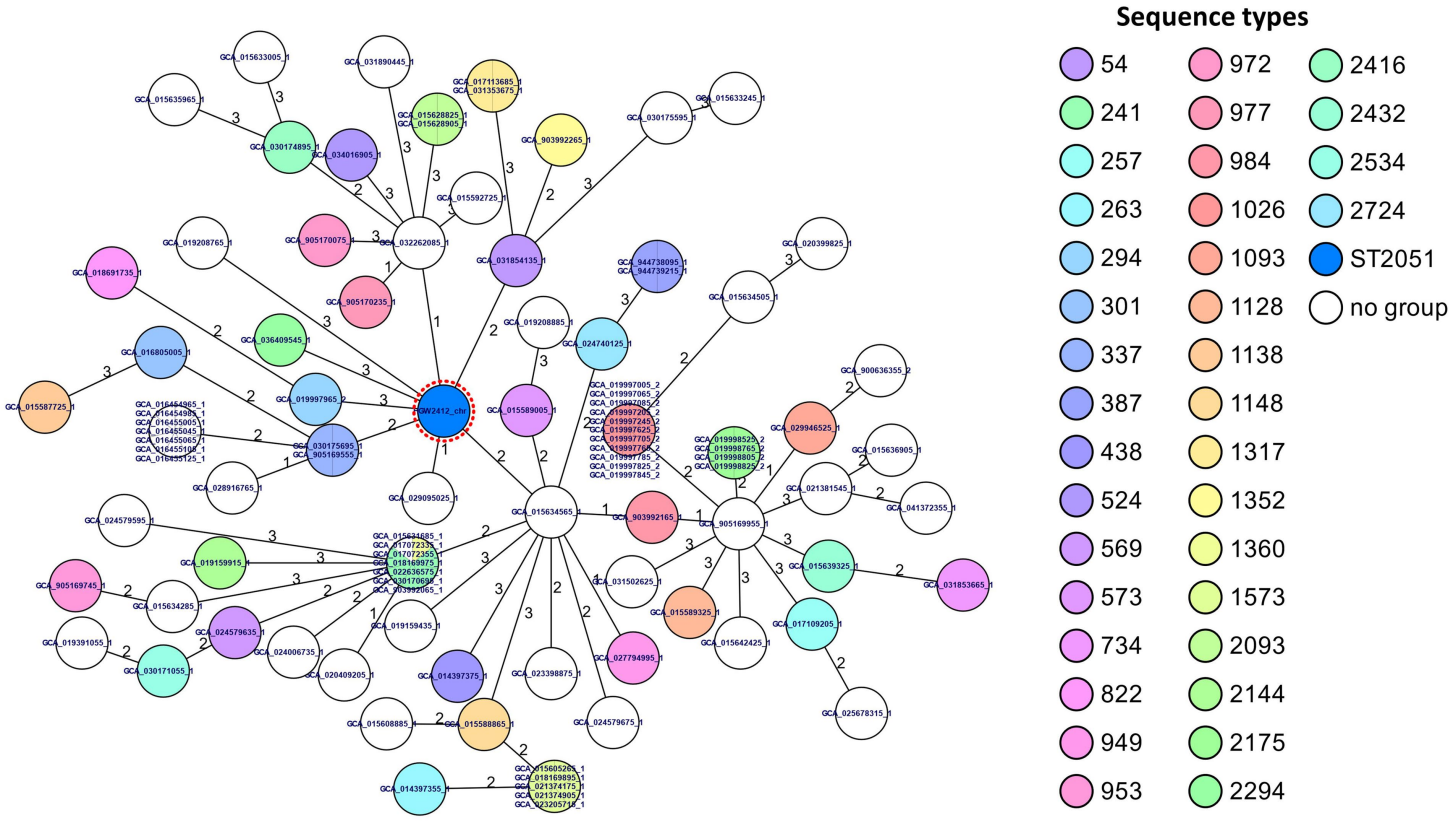
Minimum spanning tree based on MLST analysis of *S. pseudintermedius* HGW2412 and its 100 closest sequences. Sequence types (STs) were assigned using the MLST scheme for *S. pseudintermedius* implemented in the SeqSphere+ software (Ridom, Germany), applying the “pairwise ignoring missing values” option for allele comparisons. “No group” refers to new or unknown STs that could not be classified and are placed in a separate group. For clarity, the Greifswald isolate HGW2412 (HGW2412_chr) is highlighted with a dashed red circle.

For phylogenetic classification, our isolate was compared with 5,500 *S. pseudintermedius* genomes from the NCBI RefSeq database, identifying GCA_905169555 as the closest related genome using FastANI (Supplementary Table S1) (Jain et al., 2018). The 100 most similar genomes and our isolate were assigned to Multilocus Sequence Typing (MLST) (Solyman et al., 2013), which were visualized in a minimum spanning tree (Figure 4). Notably, none of the 100 closest genomes shared the sequence type (ST2051) of HGW2412, indicating high diversity. The most similar isolate GCA_905169555 differed by two housekeeping alleles, placing it in close proximity to our isolate (Figure 4). However, only one other ST2051 isolate was identified in the PubMLST database, derived from a cat in Wrocław (Poland) in 2017, but unfortunately, no associated whole-genome sequencing data are available.

**Figure 5:**
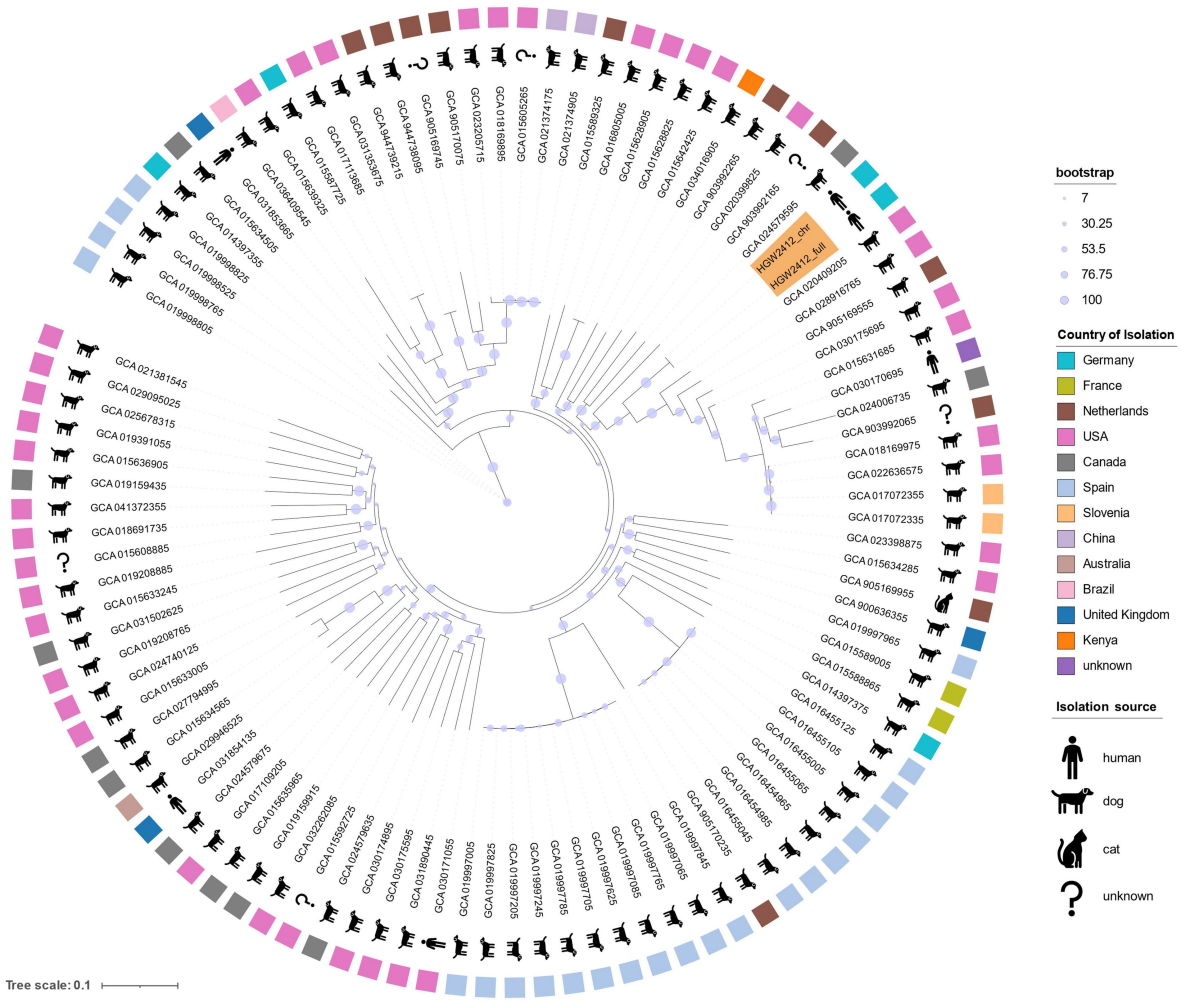
Maximum likelihood (ML) tree illustrating the phylogenetic placement of *S. pseudintermedius* HGW2412 relative to its 100 closest genomes based on core-SNP analysis. The tree is unrooted, and available metadata, including isolation source and country of origin, are annotated. Metadata were obtained from NCBI RefSeq (isolation source, country of isolation). The Greifswald isolate was included in two forms, complete genome (HGW2412_full) and chromosome only (HGW2412_chr), and is highlighted in orange. The figure was generated using Interactive Tree Of Life (iTOL v7.1.1; https://itol.embl.de/).

As no core-genome MLST scheme is available for *S. pseudintermedius*, phylogeny was reconstructed using a core-SNP approach, and the resulting tree was annotated with host and geographic metadata (Figure 5). Results revealed extensive genomic diversity and global distribution, hindering precise phylogenetic placement. HGW2412 clustered with isolates from North America, the Netherlands, Canada, Slovenia, and Kenya; German isolates appeared more distantly related. These findings were consistent with the MLST-based Minimum Spanning Tree (MST) (Figure 4), highlighting the substantial genetic heterogeneity even among the 100 most similar genomes.

### Genomic AMR gene profiling in *S. pseudintermedius*

To contextualize the AMR gene repertoire of our isolate HGW2412 within the genetically diverse species *S. pseudintermedius*, we compared it with all RefSeq genomes available in June 2025 (*n* = 5,500). In total, 34 AMR genes were identified (Figure 2A and Supplementary Table S2 – results ABRicates/heatmap_5,501 sequences) and assigned to functional gene families. Thirteen genes were associated with aminoglycoside resistance; seven, including regulatory genes, with *β*-lactam inactivation; four with macrolide resistance; three with tetracycline resistance; two with phenicol resistance; and one gene each with resistance to fosfomycin, fusidic acid, trimethoprim, mupirocin, and oxazolidinones. Aminoglycoside resistance was dominated by *aph*-Stph (99.7%), while *ant6*, *aph*(3″)-III, and *sat* were detected in approximately 40% of genomes, and *aac6*-*aph2* and *aph2* in about 30%. Among *β*-lactam resistance genes, *blaZ* (78.4%), *mecA* (39.5%), and *bla*TEM-116 (0.14%, *n* = 8) were identified. MLS resistance genes included *erm* (42.3%) and *mefA* (27.0%), whereas *lnu* and *lsaE* were rare (2.4 and 0.8%). Tetracycline resistance was mainly associated with *tetM* or *tetO* (45.5%), while *tetK* or *tetL* occurred in 6.8%. Phenicol resistance genes included *cat* (14.4%) and *fexA* (0.4%). Dihydrofolate reductase genes (*dfr*) (trimethoprim resistance) were present in 37% of genomes. All remaining AMR genes occurred in fewer than 1% of isolates (Figure 2A and Supplementary Table S2 – heatmap_5,501 sequences).

Cluster (cl) analysis of AMR gene prevalence revealed three major groups (Figure 2B and Supplementary Table S2 – SOTA_cl:1 – SOTA _cl:10). Group 1 (clusters 1–2) exhibited a multidrug-resistant gene profile, with six aminoglycoside resistance genes present in >98% of genomes, high frequencies of *blaZ* (>98%) and *mecA* (100% in cluster 1, 83.2% in cluster 2), and *erm* genes in 98.4%. *dfr* genes were also common (>97% in cluster 1; 82.3% in cluster 2). Tetracycline resistance was dominated by *tetM*/*tetO* in cluster 2 (>91%), whereas *tetK*/*tetL* were more frequent in cluster 1 (33.8%). The *mecA* regulators *mecI* and *mecR* were detected exclusively in cluster 1. Group 2 (clusters 3–7) was characterized by the generally low prevalence of *aac6-aph2* and the *mecA* regulatory genes *mecI* and *mecR*. Clusters 3–5 also showed low levels of *blaI*/*blaR*, and *blaZ* was absent in clusters 4 and 5. Our isolate HGW2412 belonged to cluster 7, which displayed high prevalences of *ant6*, *aph*(3″)-III, *aph*-Stph, and *sat* (99.7, 99.0, 100.0, and 99.0%), as well as *blaI*, *blaR*, *blaZ*, and *erm* (96.2, 85.3, 94.9, and 91.1%, respectively). Approximately 30% of cluster 7 genomes carried tetM/tetO, and nearly 60% carried the cat gene, which was also found in HGW2412. Group 3 (clusters 8–10) contained isolates with few AMR genes. Cluster 8, the largest cluster (*n* = 2,059; 37.4%), harbored *aph*-Stph (99.7%), the *blaIRZ* locus, and ~30% *mefA*, *tetM*, or *tetO*. Cluster 9 exhibited the lowest AMR gene content, containing only *aph*-Stph (100%) despite representing 13.3% of all genomes (*n* = 732). Cluster 10 resembled cluster 9 but showed higher prevalences of *blaI* (26.8%), *blaZ* (25.7%), and *mefA* (100%). In sum, a total of 42.5% (*n* = 2,337) of isolates (clusters 1–7) had gene combinations that predicted resistance to aminoglycosides, β-lactams, macrolides, sulfonamides/trimethoprim, and/or tetracyclines. By contrast, most genomes (57.5%, *n* = 3,164) harbored only a limited number of AMR genes, primarily predicting resistance to aminoglycosides, β-lactams, and/or macrolides, and were represented largely by clusters 8–10. Notably, the *mecA* gene was rarely detected in clusters 8–10. Finally, the most frequent AMR gene combinations were analyzed and computed for each cluster and are summarized in Table 2 and Supplementary Table S2. As expected, these patterns mirrored the overall AMR gene distribution; however, they also revealed substantial heterogeneity and a broad range of possible AMR gene constellations, particularly within clusters 1–7.

**Table 2:** Most common AMR gene combinations in *S. pseudintermedius.*

Cluster	Total number of AMR gene combinations within cluster	Isolates of the predominant AMR gene combination	Proportion of the predominant AMR gene combination within the cluster [%]	Proportion of total (*n* = 5,501) [%]	Predominant AMR gene combination	Theoretical resistance to some
Cluster 1	65	92	29.9	1.7	*aac6-Aph2, ant6, aph2, aph(3″)-III, apH-Stph, sat, blaI, blaR, blaZ, mecI, mecR, mecA, erm, dfr*	Aminoglycosides, β-Lactams, Macrolides, Sulfonamides/Trimethoprim
Cluster 2	163	200	17.2	3.6	*aac6-Aph2, ant6, aph2, aph(3″)-III, apH-Stph, sat, blaI, blaR, blaZ, mecA, erm, tetM/O, dfr*	Aminoglycosides, β-Lactams, Macrolides, Tetracyclines, Sulfonamides/Trimethoprim
Cluster 3	29	15	27.3	0.3	*ant6, aph(3″)-III, apH-Stph, sat, blaZ, mecA, erm, tetM/O, dfr*	Aminoglycosides, β-Lactams, Macrolides, Tetracyclines, Sulfonamides/Trimethoprim
Cluster 4	25	19	24.7	0.3	*ant6, aph(3″)-III, apH-Stph, sat, mecA, erm, tetM/O, dfr*	Aminoglycosides, β-Lactams, Macrolides, Tetracyclines, Sulfonamides/Trimethoprim
Cluster 5	32	19	23.5	0.3	*ant6, aph(3″)-III, apH-Stph, sat, blaI, mecA, erm, tetM/O, dfr*	Aminoglycosides, β-Lactams, Macrolides, Tetracyclines, Sulfonamides/Trimethoprim
Cluster 6	71	51	15.1	0.9	*ant6, aph(3″)-III, apH-Stph, sat, blaI, blaZ, mecA, erm, tetM/O, dfr*	Aminoglycosides, β-Lactams, Macrolides, Tetracyclines, Sulfonamides/Trimethoprim
Cluster 7	79	77	24.6	1.4	*ant6, aph(3″)-III, apH-Stph, sat, blaI, blaR, blaZ, erm, cat*	Aminoglycosides, β-Lactams, Macrolides, Phenicols
Cluster 8	140	724	35.2	13.2	*apH-Stph, blaI, blaR, blaZ*	Aminoglycosides, β-Lactams
Cluster 9	36	512	69.9	9.3	*apH-Stph*	Aminoglycosides
Cluster 10	37	213	57.1	3.9	*apH-Stph, mefA*	Aminoglycosides, Macrolides

## Discussion

This case illustrates the infectious potential of *S. pseudintermedius*, a species traditionally associated with veterinary medicine but increasingly reported as a human pathogen, particularly in immunocompromised individuals (Pomba et al., 2017). According to the recent case report/overview by Jones et al. (2025), *S. pseudintermedius* has been implicated in a wide range of clinical manifestations, including skin and soft tissue infections, wound and postoperative wound infections, dog-bite associated infections, pneumonia, rhinosinusitis and otitis, bacteremia, and even infections of internal organs, such as the gastrointestinal tract, joints, bones, and prosthetic implants, underscoring its growing clinical importance. Although human cases were long considered sporadic, both recent and earlier case reports indicate that *S. pseudintermedius* infections may occur more regularly and perhaps more often go unrecognized than previously assumed, particularly among individuals in close contact with companion animals such as dog owners and veterinarians (Somayaji et al., 2016; Jones et al., 2025). Interestingly, in this case, it is not known how the patient encountered *S. pseudintermedius.* Although the patient had no documented animal contact, indirect transmission via environmental surfaces, healthcare settings, or unrecognized community reservoirs cannot be ruled out, highlighting the need for broader surveillance of *S. pseudintermedius* in non-animal settings. We suspect that transmission of the pathogen occurred in the hospital, as early signs of infection were already detectable during hospitalization. Very recently, *S. pseudintermedius* was isolated from the nasal cavity of a healthcare workers and hospital environmental surfaces, a finding that lends further support to our hypothesis (Besharati et al., 2025).

Before the taxonomic separation of *S. pseudintermedius* from *S. intermedius*, the enterotoxigenic potential of *S. intermedius*, posing public health risks such as outbreaks, had already been documented (Becker et al., 2001). Its virulence factors include cytotoxins, exfoliative toxins, superantigens, and cell wall–associated proteins, which play key roles in the initiation and spread of infections, particularly skin and soft tissue infections and in evading host immune responses (Bannoehr et al., 2011; Pitchenin et al., 2018). Additionally, *S. pseudintermedius* is capable of forming biofilms, significantly enhancing its resistance and persistence in clinical settings (Teixeira et al., 2024). Enzymatic virulence mechanisms, such as proteases and thermonucleases, further contribute to pathogenicity, with plasma coagulation mediated by von Willebrand factor binding protein emerging as a particularly important factor (Pickering et al., 2021). The comprehensive WGS analysis of HGW2414 revealed a wide array of these diverse virulence factors (Supplementary Table S1). Interestingly, many of these factors share orthologs not only with *S. aureus*, but also with *S. intermedius*, *S. haemolyticus*, and *S. epidermidis*, suggesting evolutionary parallels in their pathogenic strategies (Bannoehr et al., 2011; Myrenas et al., 2024; Glajzner et al., 2023). These findings indicate that the virulence of *S. pseudintermedius* may not be exclusively linked to zoonotic infections, emphasizing its potential significance in human disease.

Whole-genome sequencing further enabled the identification of genetic determinants responsible for the observed antibiotic resistance, which partially correlated with phenotypic antimicrobial susceptibility testing (AST) results. Our isolates harbored *blaZ* resistance genes, which confer resistance to penicillin but not oxacillin. As expected, they were phenotypically methicillin-susceptible, which ultimately facilitated effective treatment and the patient’s recovery. Interestingly, genetic determinants associated with aminoglycoside and chloramphenicol resistance were detected; however, phenotypic resistance to gentamicin was not observed, highlighting the necessity of conventional AST. As mentioned above, our isolate HGW2414 was methicillin-susceptible, yet the increasing prevalence of MRSP markedly complicates the management of infections caused by this species in both veterinary and human medicine (Besharati et al., 2025; Monteiro et al., 2025). Unfortunately, research on antimicrobial-resistant *S. pseudintermedius* has predominantly focused on animal isolates, with limited reports on human cases involving MRSP (Paul et al., 2011). Our global genomic analysis of 5,501 *S. pseudintermedius* sequences confirms the frequent occurrence of multidrug-resistant strains (Figure 2 and Supplementary Table S2), theoretically resistant to aminoglycosides/streptothricin, *β*-lactams, macrolides/lincosamides/streptogramin, tetracyclines, sulfonamides/trimethoprim, and/or phenicols. Some of these putatively multidrug-resistant isolates also carried additional resistance determinants against mupirocin, fosfomycin, fusidic acid, or oxazolidinones, although these were rare. Overall, the analyzed genomes were grouped into three major groups comprising ten distinct AMR gene clusters, within which the resistance patterns were distributed. Overall, 42.5% (cluster 1–7) of all analyzed genomes displayed this multidrug-resistant profile, underscoring the clinical and epidemiological significance of such isolates within the species. In contrast, 57.5% (cluster 8–10) of isolates harbored only a limited number of AMR genes, suggesting that they could theoretically be treated with older, well-established antibiotics, potentially preventing further resistance acquisition. Our genomic AMR findings are broadly consistent with previous studies on antibiotic resistance in *S. pseudintermedius* (Tyson et al., 2021), although earlier reports often focused on phenotypic resistance rather than AMR gene prevalence (Besharati et al., 2025; Monteiro et al., 2025; Nocera and De Martino, 2024; Morais et al., 2023). Notably, Monteiro et al. (2025) reported a high proportion of fluoroquinolone-resistant isolates, whereas in our dataset we detected, e.g., no plasmid-mediated fluoroquinolone resistance genes, such as *qnr* or *aac*(6′)-*Ib*-*cr*. This likely reflects the well-described mechanism in staphylococci, in which point mutations in the target genes *gyrA* (DNA gyrase subunit A) and *grlA* (topoisomerase IV subunit A) confer fluoroquinolone resistance, a mechanism that was not assessed in our analysis (Tyson et al., 2021; Morais et al., 2023). Cluster analyses revealed patterns of AMR gene accumulation in specific isolates, potentially linked to other genetic determinants, such as the frequent occurrence of MRSP among particular sequence types (Besharati et al., 2025; Nocera and De Martino, 2024). Both, our data and previous reports highlight the high prevalence of *mecA*-positive *S. pseudintermedius* isolates, reinforcing the need for systematic AMR screening of *S. pseudintermedius* in animal and human medicine. Such surveillance is essential to optimize therapy, avoid unnecessary antibiotic use, and mitigate the spread of multidrug-resistant strains among zoonotic pathogens. Thus, the multidrug-resistant profile of MRSP and, to a lesser extent MSSP, especially regarding resistance to multiple key antibiotic classes, underscores the need for strong antimicrobial stewardship, informed by both phenotypic and genomic resistance testing, even in MSSP (Kadlec and Schwarz, 2012).

Since the source of infection could not be identified, phylogenetic analyses may aid in the spatial classification of the isolates. Advances in molecular typing have greatly enhanced our understanding of the SIG species (Bannoehr et al., 2007), but a specific MLST scheme for *S. pseudintermedius* was only introduced in 2013, accompanied by the establishment of a public database to improve surveillance of this species (Solyman et al., 2013). However, *S. pseudintermedius* exhibits substantial clonal diversity, which complicates precise epidemiological surveillance using MLST (Morais et al., 2023; Grist et al., 2025). While certain sequence types (e.g., ST45, ST71, ST258) are more prevalent, genetic diversity remains remarkably high even within a single geographic region or same isolation hosts (Solyman et al., 2013; Morais et al., 2023; Grist et al., 2025; Damborg et al., 2016; Videla et al., 2018). This was also evident in our analysis, where even those genomes identified as highly similar by FastANI differed by at least two alleles in the MLST scheme (Figure 4). We identified sequence type ST2051 for the HGW2412 isolate, a ST previously found in Poland. It is possible that both isolates are genetically closely related, a hypothesis supported by their geographic proximity; however, no WGS data of the polish isolate were available to confirm this. To address this epidemiological and diagnostic limitation, WGS combined with core genome MLST (cgMLST) enables high-resolution genotyping through genome-wide, gene-by-gene allele calling of conserved loci. This approach is recombination-robust, standardized, and scalable, making it ideally suited for routine laboratory use, including the surveillance of multidrug-resistant bacteria (Mellmann et al., 2016). To date, no cgMLST scheme exists for *S. pseudintermedius*, likely due to its high genetic diversity, which poses challenges for standardized scheme development. However, establishing such a scheme would be highly desirable, particularly in the context of the “One Health” frame work. Our core-SNP analysis confirmed the MLST results (Figure 4) and highlighted substantial genetic variability, which impeded precise phylogenetic resolution (Figure 5). The effectiveness of the core-SNP approach became particularly evident in the identification of closely related sequences, especially those from Spain, as well as from Slovenia and the USA (Figure 5); yet a clear geographic association could not be established based on these results. However, in addition to molecular-based methods, MALDI-TOF MS has made significant advances and greatly facilitated the species-level identification of SIG members (Canver et al., 2019; Decristophoris et al., 2011), although it is not suitable for resolving epidemiological questions.

## Conclusion

To support ongoing surveillance and research, the development of novel, accurate epidemiological tools, such as an internationally standardized cgMLST scheme for *S. pseudintermedius*, is essential. This scheme should be systematically validated across both animal and human *S. pseudintermedius* isolates to ensure its efficacy in One Health-based AMR gene surveillance. Equally important is the rapid and reliable identification of *S. pseudintermedius* in clinical settings (human and animal!), as timely recognition of the pathogen is critical not only for initiating targeted antimicrobial therapy and improving clinical outcomes, but also for preventing further spread and limiting the development of additional multidrug resistance.

## Data Availability

The datasets presented in this study can be found in online repositories. The names of the repository/repositories and accession number(s) can be found at: https://www.ncbi.nlm.nih.gov/, PRJNA1260423.

## Footnotes

1
https://github.com/nanoporetech/dorado

2
https://github.com/nanoporetech/medaka

3
https://github.com/nmquijada/tormes

4
https://github.com/oschwengers/bakta

5
https://www.mgc.ac.cn/VFs/main.htm

6
https://github.com/enormandeau/ncbi_blast_tutorial

7
https://www.ncbi.nlm.nih.gov/refseq/

8
https://github.com/ParBLiSS/FastANI?tab=readme-ov-file

9
https://www.ncbi.nlm.nih.gov/datasets/genome/?taxon=283734

10
https://github.com/tseemann/abricate
